# A SNAP-Tagged Derivative of HIV-1—A Versatile Tool to Study Virus-Cell Interactions

**DOI:** 10.1371/journal.pone.0022007

**Published:** 2011-07-22

**Authors:** Manon Eckhardt, Maria Anders, Walter Muranyi, Mike Heilemann, Jacomine Krijnse-Locker, Barbara Müller

**Affiliations:** 1 Department of Infectious Diseases, Virology, University Hospital Heidelberg, Heidelberg, Germany; 2 BIOQUANT Centre, University of Heidelberg, Heidelberg, Germany; 3 Biotechnology and Biophysics, Julius-Maximilians-University Würzburg, Würzburg, Germany; 4 Electron Microscopy Core Facility, BIOQUANT, University of Heidelberg, Heidelberg, Germany; University of Pittsburgh, United States of America

## Abstract

Fluorescently labeled human immunodeficiency virus (HIV) derivatives, combined with the use of advanced fluorescence microscopy techniques, allow the direct visualization of dynamic events and individual steps in the viral life cycle. HIV proteins tagged with fluorescent proteins (FPs) have been successfully used for live-cell imaging analyses of HIV-cell interactions. However, FPs display limitations with respect to their physicochemical properties, and their maturation kinetics. Furthermore, several independent FP-tagged constructs have to be cloned and characterized in order to obtain spectral variations suitable for multi-color imaging setups. In contrast, the so-called SNAP-tag represents a genetically encoded non-fluorescent tag which mediates specific covalent coupling to fluorescent substrate molecules in a self-labeling reaction. Fusion of the SNAP-tag to the protein of interest allows specific labeling of the fusion protein with a variety of synthetic dyes, thereby offering enhanced flexibility for fluorescence imaging approaches.

Here we describe the construction and characterization of the HIV derivative HIV^SNAP^, which carries the SNAP-tag as an additional domain within the viral structural polyprotein Gag. Introduction of the tag close to the C-terminus of the matrix domain of Gag did not interfere with particle assembly, release or proteolytic virus maturation. The modified virions were infectious and could be propagated in tissue culture, albeit with reduced replication capacity. Insertion of the SNAP domain within Gag allowed specific staining of the viral polyprotein in the context of virus producing cells using a SNAP reactive dye as well as the visualization of individual virions and viral budding sites by stochastic optical reconstruction microscopy. Thus, HIV^SNAP^ represents a versatile tool which expands the possibilities for the analysis of HIV-cell interactions using live cell imaging and sub-diffraction fluorescence microscopy.

## Introduction

The labeling of individual viral proteins by fusion to fluorescent molecules in conjunction with advanced fluorescence imaging techniques has greatly expanded the possibilities to investigate virus-cell interactions. This includes live-cell imaging approaches to study the dynamics of intracellular events as well as super-resolution fluorescence microscopy methods surmounting the diffraction barrier of light microscopy, which allow the analysis of fluorescently labeled structures at a resolution down to ∼20 nm (for review see [1], [2]). Human immunodeficiency virus (HIV) derivatives labeled by fusion of fluorescent proteins (FPs) to the structural polyprotein Gag, the accessory protein Vpr or the viral integrase, respectively, have been successfully employed to analyze cell entry as well as particle assembly of HIV by live cell fluorescence microscopy (e.g. [3]–[11], reviewed in [2], [12]). Sub-diffraction microscopy has been employed in proof of principle studies to display the distribution and mobility of HIV-1 Gag molecules at the plasma membrane of virus producing cells [13], [14]. While FPs have become invaluable tools in cell biology and virology, some of their properties present disadvantages which limit their usefulness in live-cell microscopy: (i) FPs are inferior to many modern synthetic fluorophores with respect to quantum yield and photostability, which restricts time resolution and the duration of observation in live-cell experiments. (ii) Although a continuously increasing range of FPs with different spectral properties is available [15], the color range is limited, in particular in the blue and far-red range. Only few FPs display the photophysical properties rendering them suitable for sub-diffraction microscopy methods. (iii) The fluorophores of FP display relatively slow maturation kinetics [16]; consequently, newly expressed FP molecules are initially undetectable by fluorescence microscopy, which limits their use in pulse-chase experiments. (iv) Some FPs are obligatory multimers, which may affect the functionality of the cellular fusion partner. (v) Finally, experimental setups in cell biology often involve multi-color approaches using several differentially labeled proteins. In the case of FP fusion proteins, individual expression constructs have to be cloned and characterized in order to obtain different spectral variants of a protein of interest. Genetically encoded non-fluorescent labels which can be specifically stained using synthetic fluorescent dyes offer a greater flexibility in the choice of label. A well-known example of this type of motifs is the six to twelve amino acid long tetracysteine (TC)-tag [17], which is attractive due to its small size. The TC-tag has been employed for the generation of tagged HIV derivatives [18]–[20]; however, disadvantages of this system include a high degree of intracellular background staining [21], [22], the dependency of staining from the redox state of the tag [17] and a very limited selection of compatible fluorescent dyes. We therefore decided to explore the use of the so-called SNAP-tag for labeling of HIV. The SNAP-tag is a genetically encoded label with a slightly lower molecular mass than FPs (∼20 kDa), which is derived from of the mammalian DNA repair protein O^6^-alkylguanin-DNA-alkyltransferase. It stably transfers the benzyl moiety of O^6^-benzylguanine (BG) substrates to its active site [23] and thereby performs a self-labeling reaction when fluorescently labeled derivatives of BG are offered as substrate [24]. Analogous to FP encoding sequences, the SNAP-tag coding sequence can be genetically fused to the coding sequence of any protein of interest. The resulting fusion protein is non-fluorescent but can be labeled upon addition of the substrate of choice. Available BG coupled dyes represent a wide variety of synthetic fluorophores with good quantum yield and photostability. As the reactivity of the SNAP-tag is largely independent of the nature of the attached fluorescent probe [24], any available synthetic dye coupled to BG can in principle be used in this setting. Here, we describe the generation and characterization of an HIV derivative which carries a SNAP-tag within the main structural polyprotein Gag. HIV Gag is necessary and sufficient for formation and release of virus like particles from expressing cells [25]. During the HIV assembly process, Gag orchestrates the incorporation of other virion components and interacts with cellular factors required for particle release. The fate of Gag during the replication cycle of HIV has been studied by a variety of methods (for review see e.g. [26]–[28]). However, further elucidation especially of the dynamic interactions with host cellular proteins will be of major interest. The SNAP-tagged HIV derivative (HIV^SNAP^) established in this study was functional with respect to virus-cell fusion and particle assembly and allowed virus replication in tissue culture. Gag.SNAP could be specifically stained in the viral and cellular context. HIV^SNAP^ thus represents a versatile tool to study HIV-cell interactions.

## Results and Discussion

**Figure 1:**
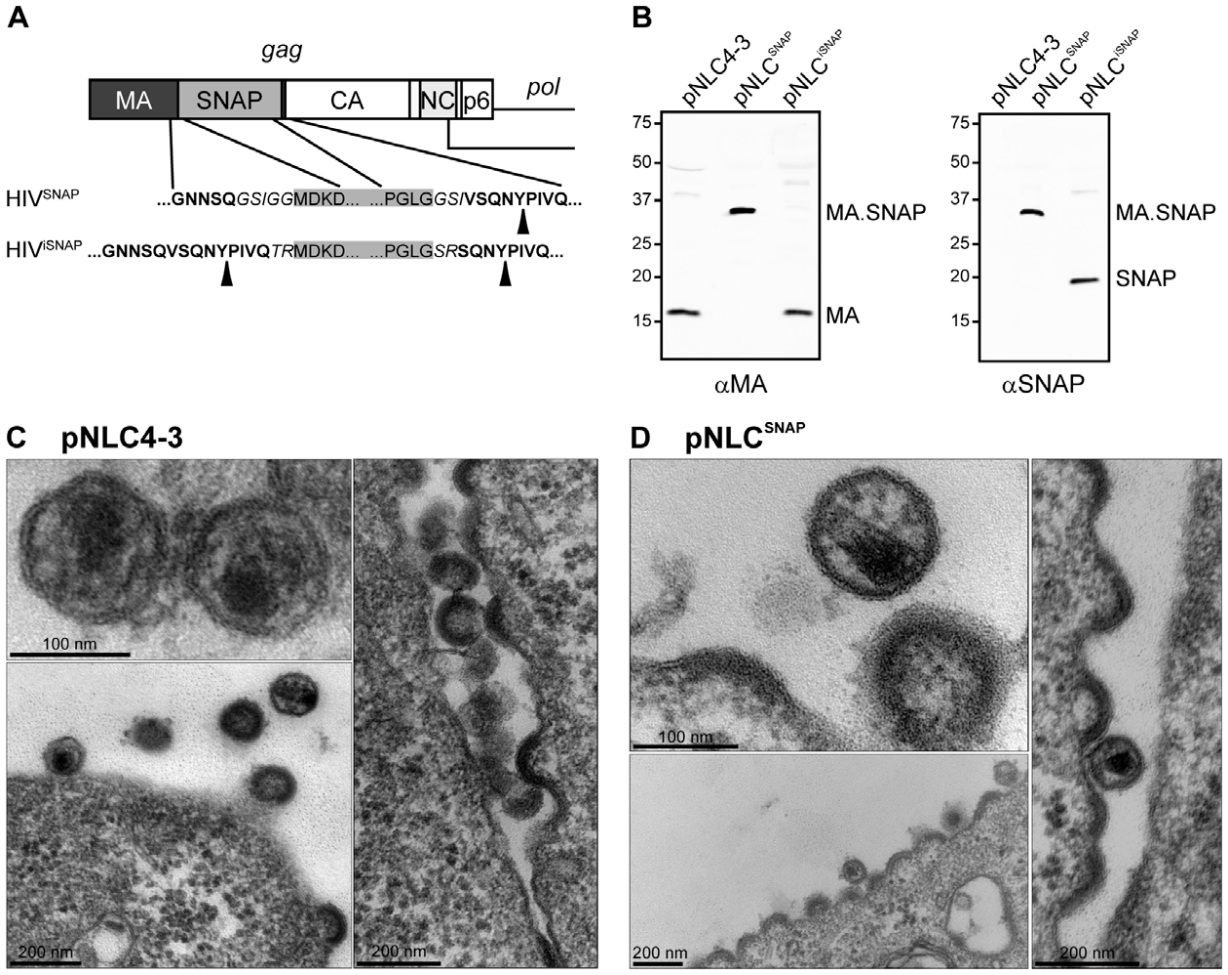
Construction and characterization of an HIV derivative carrying a SNAP-tag within Gag. (**A**) Scheme of the HIV-1 *gag* open reading frame indicating the *snap-tag* gene inserted between the MA and CA coding regions. Expanded regions display the junction between Gag (bold) and SNAP-tag (grey boxes) amino acid sequences, separated by short linker sequences (italics) for HIV^SNAP^ and HIV^iSNAP^ constructs, respectively. Arrowheads indicate cleavage sites for HIV-1 PR. (**B**) Immunoblot analysis of wt and modified virions. Particles released into the supernatant of 293T cells transfected with the indicated proviral plasmids were purified by ultracentrifugation and subjected to immunoblot analysis using the indicated antisera as described in methods. Positions of molecular mass standards are indicated at the left (in kDa). (**C**, **D**) Morphology of HIV and HIV^SNAP^ virions. 293T cells transfected with pNLC4-3 (C) or pNLC^SNAP^ (D), respectively, were fixed at 24 h post transfection and embedded for EM analysis as described in methods. Samples were analyzed by thin section electron microscopy.

**Figure 2:**
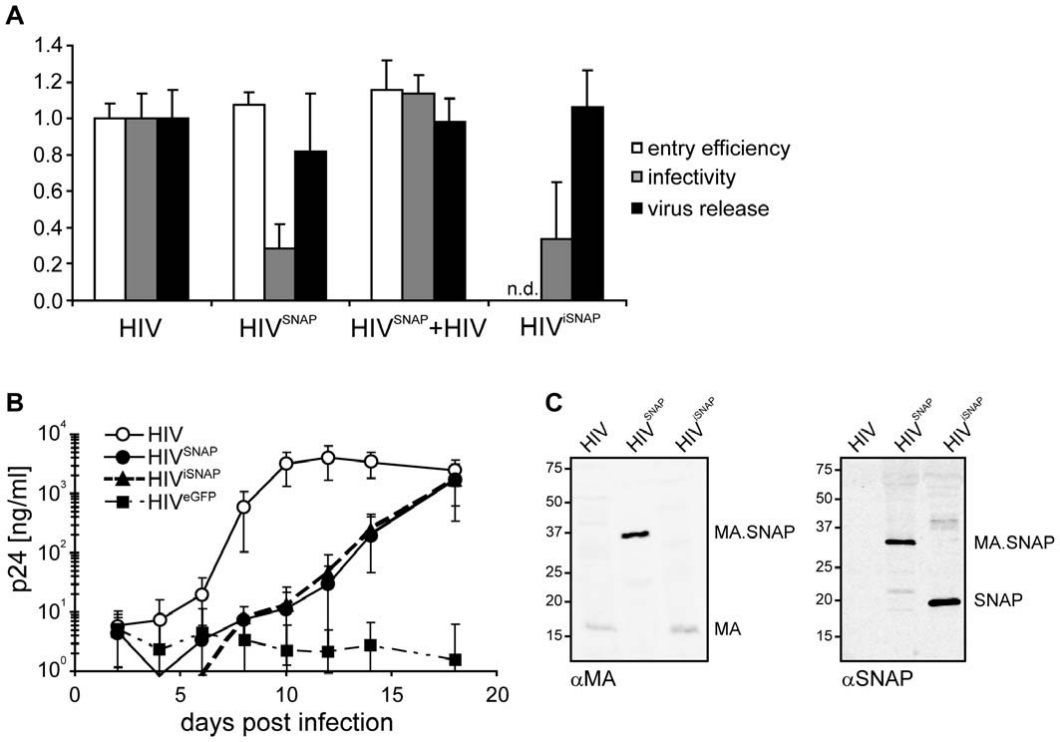
Replication capacity of SNAP-tagged HIV derivatives. (**A**) Influence of the SNAP-tag insertion on distinct steps in HIV replication. 293T cells were transfected with pCHIV or pNLC4-3 (HIV), pCHIV^SNAP^ or pNLC^SNAP^ (HIV^SNAP^), an eqimolar mixture of both plasmids (HIV^SNAP^+HIV), or with pNLC^iSNAP^ (HIV^iSNAP^), respectively. Virions were harvested from the supernatant at 40 h post transfection. Values were normalized against the mean of the respective control. White bars: Entry efficiency of pCHIV-derived particles, purified by ultracentrifugation through a sucrose cushion, was measured on JC53 cells using the β-lactamase virion fusion assay as described in methods. Mean values and standard deviation from triplicate samples are shown. n.d. = not determined. Light grey bars: Infectivity (in r.l.u. per ng CA) of pNLC4-3-derived particles was determined by titration of viurs supernatants on TZM-bl reporter cells followed by analysis of luciferase activity as described in methods. Bars represent mean values and standard deviation from 6 independent experiments. Black bars: For determination of release efficiency, the amount of p24 CA released into the supernatant at 40 h post transfection was measured by quantitative immunoblot and divided by the sum of p24 in the supernatant and cell lysates. Mean values and standard deviations from three independent experiments are shown. (**B**) Replication capacity of HIV derivatives. Equal amounts of virus (0.5 ng p24 equivalent) produced from 293T cells transfected with pNLC4-3 (HIV; open circles), pNLC^SNAP^ (HIV^SNAP^; filled circles), pNLC^iSNAP^ (HIV^iSNAP^; filled triangles), or pNLC^eGFP^ (HIV^eGFP^; filled squares), respectively, were used to infect MT-4 cells. At the indicated time points post infection the amount of CA released into the supernatant was determined by a quantitative dot blot analysis. Mean values and standard deviations from six replicate infections are shown. (**C**) Retention of the SNAP-tag after several rounds of replication. Particles from tissue culture supernatants of infected MT-4 cells at day 18 post transfection were analyzed by immunoblot using the indicated antisera. The position of molecular mass standards is indicated at the left (in kDa).

For introduction of the SNAP-tag into the HIV structural polyprotein Gag we made use of the fact that Gag is a modular protein, consisting of individually folded subdomains separated by flexible linker regions [29]. Proteolytical processing by the viral protease (PR) at specific sites within these linker regions triggers morphologic rearrangements of the mature Gag subunits matrix (MA), capsid (CA), nucleocapsid (NC) and p6 within the virion, which are essential for virion infectivity. Cryo electron microscopy had shown that within the immature virion, Gag molecules are arranged in a parallel manner underneath the viral membrane, and the region separating the globular MA and CA domains of Gag forms a layer of low protein density [30]. We have previously demonstrated that foreign amino acid sequences can be inserted within this region without affecting Gag expression, assembly or proteolytic maturation, and that small insertions are tolerated without affecting virus replication in tissue culture [31]; this has been exploited for the construction of various modified HIV derivatives by us and others [4], [19], [31]–[33]. Based on this, we cloned the SNAP-tag coding sequence between the codons for amino acids 128 and 129 of MA as outlined in Figure 1 A. Four C-terminal residues of MA were retained C-terminally of SNAP in order to allow efficient processing downstream of MA.SNAP by HIV PR (HIV^SNAP^, Figure 1 A). The SNAP-tagged derivative was generated both in the context of an infectious HIV-1_NL4-3_ based proviral plasmid (yielding pNLC^SNAP^), as well as in the context of the non-replication competent derivative pCHIV [4], which lacks the viral long terminal repeat regions but encodes all HIV proteins except for Nef (pCHIV^SNAP^). In addition we constructed an HIV-1_NL4-3_ derivative carrying the SNAP-tag between MA and CA flanked by two PR cleavage sites (pNLC^iSNAP^; HIV^iSNAP^, Figure 1 A). In the case of eGFP labeled derivatives, the analogous construct pNLC^eGFP^ did not display robust replication in tissue culture, while the introduction of an additional PR cleavage site upstream of the eGFP domain was reported to display nearly wild-type (wt) replication kinetics at least in MT-4 T-cells [33]. Virus particles prepared by ultracentrifugation from the tissue culture supernatant of 293T cells transfected with pNLC4-3, pNLC^SNAP^ and pNLC^iSNAP^, respectively, were analyzed by immunoblot for HIV protein composition, proteolytic processing of polyproteins, as well as for the presence of SNAP-tag fusion proteins. Particles displayed the expected viral protein composition and the modified Gag.SNAP polyprotein was efficiently processed during particle maturation (Figure 1 B and data not shown). The fusion protein MA.SNAP (∼37 kDa), reactive to immunostaining with antiserum raised against HIV-1 MA as well as with anti-SNAP antiserum, was shown to be present in HIV^SNAP^ particles, whereas two separate bands, corresponding to MA (17 kDa) and SNAP (20 kDa), were detected in immunoblots of HIV^iSNAP^ samples. Electron micrographs of cells transfected with either wt (Figure 1 C) or SNAP-tagged proviral plasmids (Figure 1 D) showed viral budding profiles as well as immature and mature virions. Cells transfected with the SNAP-tagged derivative displayed a higher number of early budding structures; similar results had been obtained for an eGFP-tagged HIV derivative [31]. Since only moderately reduced amounts of virus particles were pelleted from the supernatants of HIV wt and pCHIV^SNAP^ transfected cells harvested at 40 h post transfection (see Figure 2 A, black bars), this phenotype may be explained by delayed budding kinetics, resulting in increased numbers of budding structures detected in the steady state represented in still images. Mature HIV^SNAP^ particles were found to display wt morphology (Figure 1 D).

We then characterized the functional properties of the modified virus. The FP labeled derivative HIV^eGFP^ had been shown to be infectious in tissue culture, but its infectivity was significantly reduced compared to wt HIV [31] and did not sustain multiple replication rounds in tissue culture. Co-transfection of cells with an equimolar mixture of pNLC^eGFP^ and wt pNLC4-3 results in the production of mixed particles displaying wt single-round infectivity [31]. The introduction of other foreign sequences between the MA and CA domains yielded HIV derivatives with varying levels of infectivity, ranging from wt infectivity to a complete lack of particle production ([18], [19], [31], [32] and unpublished observations). Despite a trend towards smaller insertions generally being more favorable, we did not observe a direct correlation between the molecular mass of the inserted domain and the infectivity of the modified virus in our own studies. We compared HIV^SNAP^ and HIV^iSNAP^ to the wt virus, as well as to mixed particles carrying approximately equimolar amounts of Gag and Gag.SNAP, respectively, with respect to the efficiency of individual replication steps (Figure 2 A). In order to determine the efficiency of cytoplasmatic entry, we made use of the so-called beta-lactamase virion fusion assay (BlaM assay) for HIV cell fusion [34]. This assay relies on reporter virions carrying beta-lactamase (BlaM); cytoplasmatic delivery of BlaM upon virus-cell fusion is monitored by loading the target cells with the fluorescent substrate CCF-2, which changes its fluorescence properties upon BlaM mediated cleavage. Reporter particles were prepared from the tissue culture supernatant of 293T cells co-transfected with plasmids pMM310 (coding for Vpr.BlaM) and pCHIV, pCHIV^SNAP^, or an equimolar mixture of both, respectively. Fusogenicity of particles was tested by incubation of JC53 cells with equal amounts of virus followed by CCF-2 staining and fluorimetric analysis as described in the methods section. We found that pCHIV^SNAP^ derived particles (HIV^SNAP^) displayed comparable fusion efficiency as their unlabeled counterpart (HIV) or the mixed virions (HIV^SNAP^+HIV), respectively (Figure 2 A, white bars). Virion infectivity was analyzed by incubating TZM-bl reporter cells with particles prepared from 293T cells transfected with pNLC4-3, pNLC^SNAP^, a mixture of both plasmids, or pNLC^iSNAP^, respectively. Analysis of HIV induced luciferase reporter activity at 48 h post infection revealed that HIV^SNAP^ and HIV^iSNAP^ virions were approximately 3-fold reduced in infectivity as compared to wt HIV. The subtle increase in infectivity of HIV^iSNAP^ virions compared to HIV^SNAP^ was found to be non-significant (p = 0.65 as determined by student's t-test). Full infectivity on TZM-bl cells could however be restored in mixed particles (Figure 2 A, gray bars). Since HIV^SNAP^ was not impaired in virus-cell fusion, the decreased infectivity indicates a defect at replication steps between cytoplasmatic entry and viral gene expression. The late stages of virus replication were analyzed by quantitating CA released into the supernatant relative to the total amounts of Gag expressed in transfected cells (Figure 2 A, black bars). HIV^SNAP^ displayed no significant impairment in bulk particle release (∼20% decrease; p = 0.097 as determined by student's t-test), which was not observed for HIV^iSNAP^. Again, wt properties were restored for HIV^SNAP^ upon co-transfection with equimolar amounts of wt plasmid. In order to test the replication capacity of the modified virus over multiple rounds of infection in a T-cell line, particles generated by transfection of 293T cells using pNLC4-3, pNLC^SNAP^ or pNLC^iSNAP^, respectively, were used to infect MT-4 cells and the amount of p24 CA released into the tissue culture supernatant was monitored over 18 days post infection (Figure 2 B). Replication kinetics of HIV^SNAP^ (filled circles) were significantly delayed compared to wt HIV (open circles), but comparable levels of released virus were reached at 18 days post infection. The introduction of an additional PR cleavage site between the MA and SNAP moieties (HIV^iSNAP^, triangles) did not result in increased replication capacity under these conditions. Since we did not observe a significant functional improvement of HIV^iSNAP^ compared to HIV^SNAP^ in our assays, we focused on HIV^SNAP^ for further characterization. The fact that the tag remains associated with a viral protein even after proteolytic Gag maturation is advantageous for many experimental setups, in particular for analyses of HIV entry. In contrast to the analogous construct HIV^eGFP^, which did not replicate to detectable levels in this cell line (filled squares), HIV^SNAP^ can be employed for the study of virus-cell interactions in infected cells. Immunoblot analyses of HIV^SNAP^ from the tissue culture supernatant at day 18 post infection (Figure 2 C) revealed only SNAP-tagged Gag derivatives, indicating that the modified virus was stable over several rounds of replication. These analyses were carried out using the modified virus in the absence of SNAP substrates. In order to test whether staining of Gag.SNAP affected virion infectivity, purified HIV^SNAP^ virions treated with TMR-*Star* or DMSO, respectively, were tested for infectivity by titration on TZM-bl reporter cells as described in Methods S1. Staining efficiency was analyzed in parallel by confocal microscopy of glass-bound particles after counterstaining with antiserum raised against HIV-1 CA. We found that particle preparations giving rise to approximately 86% of double-labeled virions were only moderately impaired in single-round infectivity as compared to the DMSO control virus. Wild-type HIV-1 used as a negative control was not stained by TMR*-Star*, but also displayed a similar reduction in infectivity upon TMR-Star treatment, indicating that the reduction was due to the treatment procedure rather than by the presence of MA.SNAP -TMR*-Star* (Figure S1).

**Figure 3:**
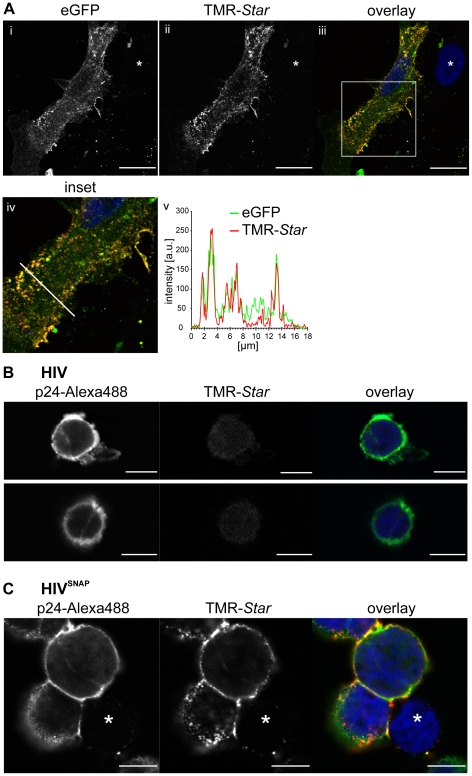
Specific intracellular staining of SNAP-tagged Gag protein. (A) Intracellular detection of SNAP-tagged Gag protein in transfected HeLa cells. HeLa cells transfected with an equimolar mixture of pCHIV^eGFP^ and pCHIV^SNAP^ were labeled at 24 h post transfection using BG-TMR-*Star* as described in methods; nuclei were stained with Hoechst 33258. The figure shows confocal images of a mid-section through a representative cell in the green channel (i), red channel (ii), and as an overlay of green, red and blue channel (iii). Scale bars: 18 µm. Pearson's coefficient for green and red channels equals 0.754. The image in (iv) represents an enlarged section from (iii). (v) Fluorescence intensity profiles from the green and red channel along the white line shown in (iv). (B, C) Intracellular detection of SNAP-tagged Gag protein in infected C8166 T-cells. C8166 cells were infected with HIV (B) or HIV^SNAP^ (C), respectively, produced from transfected 293T cells. At 15 days post infection, cells were stained with BG-TMR-*Star* as well as by immunofluorescence using an antiserum raised against HIV-1 CA; nuclei were stained with Hoechst 33258. The figure shows confocal images of mid-sections for immunofluorescence (left panels), TMR-*Star* (middle panels) and an overlay of αp24, TMR-*Star* and Hoechst signals (right panels). Scale bars: 8 µm. White asterisks: non-infected cell.

The suitability of the SNAP-tag label for specific detection of intracellular HIV Gag was demonstrated by transfection of HeLa cells with pCHIV^SNAP^ followed by staining of the transfected cells with BG-TMR-*Star* (Figure 3 A). Co-transfection with the previously characterized HIV derivative pCHIV^eGFP^ [4] which allows the efficient detection of Gag using fluorescence microscopy techniques was employed to investigate SNAP-tag staining specificity and intracellular localization of Gag.SNAP. The TMR-*Star* reactive Gag protein co-localized with Gag.eGFP in virus expressing cells (Figure 3 A iii–v), indicating that Gag.SNAP showed the correct intracellular localization and that Gag.SNAP molecules arranged in Gag assemblies are accessible to the staining procedure. Specificity of TMR-*Star* staining for transfected cells was demonstrated by lack of signal in non transfected cells (Figure 3 A, white asterisk). To further test the applicability of the tag in a more physiological context, the T-cell line C8166 was infected with wt HIV or HIV^SNAP^, respectively, and stained with the SNAP-tag reactive dye TMR-*Star* at 15 days post infection, i.e. after several rounds of virus replication. To visualize infected cells, we performed counterstaining with antiserum raised against HIV-1 CA. No unspecific staining by TMR-*Star* was detected in cells infected with wt HIV (Figure 3 B). In contrast, C8166 cells infected with HIV^SNAP^ virions displayed specific staining of Gag.SNAP by the fluorescent SNAP substrate (Figure 3 C), while staining was absent in cells not reactive to CA antiserum (Figure 3 C, white asterisk). The staining pattern of TMR-*Star* differed somewhat from the pattern revealed by immunolabeling, with the TMR-*Star* label displaying a higher number of distinct punctuate signals at the plasma membrane of virus producing cells (Figure 3 C, compare left and middle panel). This is explained by the impaired antibody accessibility of epitopes within CA in assembled viral structures, which is known to affect detection of intracellular Gag assemblies by immunostaining and immunoprecipitation approaches [35]. In contrast, assembled Gag.SNAP appeared to be accessible for specific staining, yielding a more faithful representation of intracellular Gag distribution. We conclude that HIV^SNAP^ variants are suitable for live-cell imaging analyses in infected T-cells.

**Figure 4:**
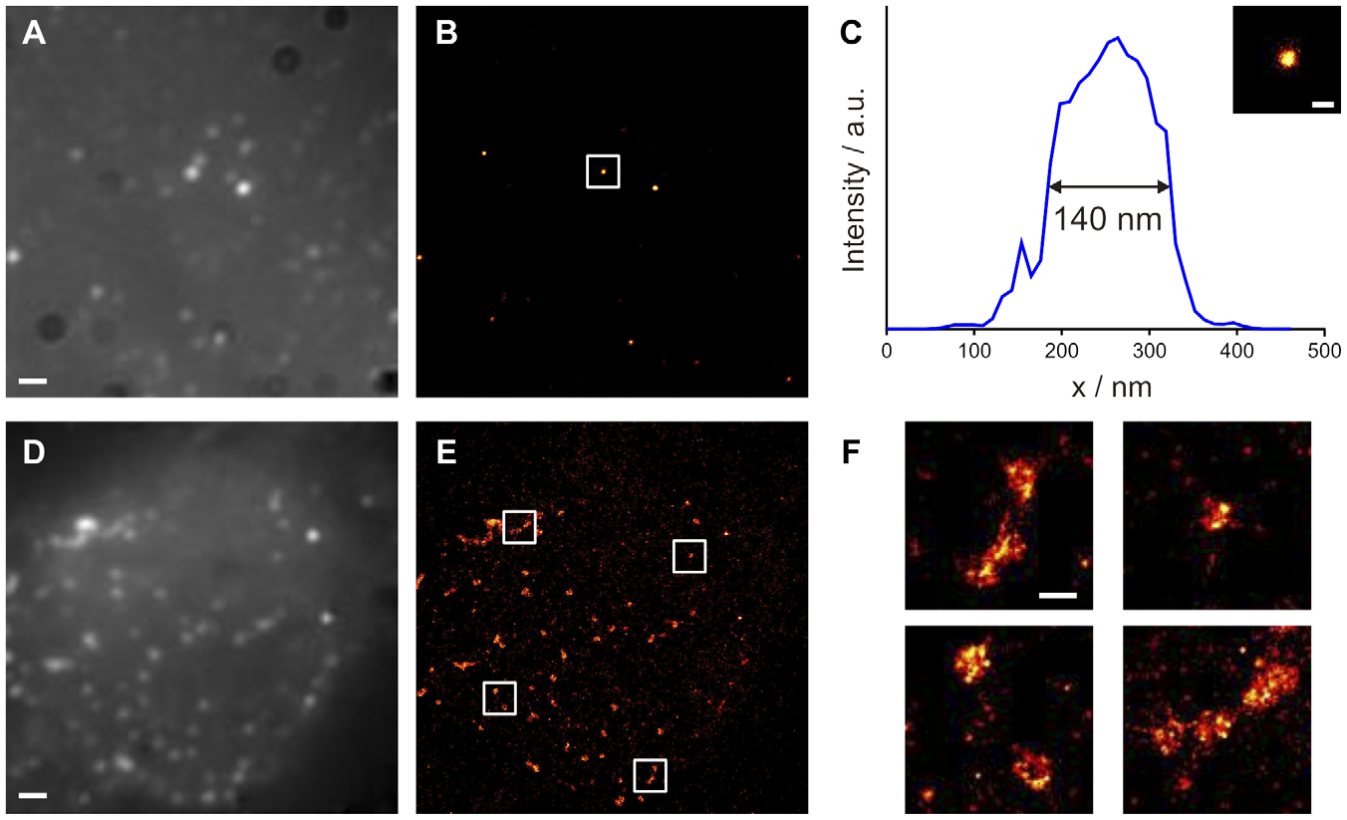
Visualization of HIV^SNAP^ virions and budding sites by super-resolution TIRF microscopy. (**A–C**) High-resolution microscopy of stained HIV^SNAP^ particles. HIV^SNAP^ particles were purified by ultracentrifugation from the supernatant of transfected 293T cells, bound to glass coverslips and stained using SNAP-surface 647. Summed-molecule TIRF (**A**) and dSTORM (**B**) fluorescence images of the same region of interest are shown. The white box indicates the particle magnified in (C). Scale bar: 1 µm. (**C**) Cross sectional profile derived from the dSTORM image of an individual HIV particle. Scale bar: 200 nm. (**D–F**) HIV budding sites at the plasma membrane of an HIV^SNAP^ expressing T-cell. A3.01 cells were nucleofected with pCHIV^SNAP^, stained with SNAP-surface 647 and imaged as described in materials and methods. Summed-molecule TIRF (**D**) and dSTORM (**E**) fluorescence images of the same cell are shown. (**F**) Magnified view of the boxed regions from the dSTORM image shown in (D). Scale bar: 200 nm.

The presence of the SNAP-tag, which can be labeled with a variety of synthetic dyes, also makes the fusion protein amenable to visualization by super-resolution fluorescence microscopy techniques. Stimulated emission depletion (STED) microscopy [36] or stochastic optical reconstruction microscopy (STORM) [37] allow the localization of fluorescent molecules and the analysis of labeled structures with a resolution well below the diffraction limit [38], given that the protein of interest can be specifically labeled with a suitable fluorophor. The genetically encoded SNAP-tag in conjunction with appropriate dyes has recently been demonstrated to be suitable for STED or STORM analyses in living cells [39], [40]. HIV^SNAP^ thus provides a basis for the analysis of sub-viral structures in living cells. As a proof of principle we applied direct STORM (dSTORM), a variation of STORM relying on light-induced reversible photoswitching of fluorophors [41], to visualize purified HIV^SNAP^ particles (Figure 4 A–C) as well as the membrane of HIV^SNAP^ expressing A3.01 T-cells (Figure 4 D–F) in TIRF (total internal reflection fluorescence) mode. As shown in Figure 4 C, dSTORM images of purified virions resolved individual punctuate signals with an average full width half maximum of ∼140 nm, which is in good agreement with the average diameter of HI virions (145 nm) determined by cryo-electron microscopy [42]. dSTORM images of the plasma membrane of HIV^SNAP^ expressing T-cells revealed individual budding sites with diameters around 150 nm; closely adjacent budding sites were resolved in the dSTORM analysis (Figure 4 E, F).

In conclusion, we consider the SNAP-tag based labeling strategy presented here a useful expansion of the existing panel of fluorescent HIV derivatives and a versatile tool to study different aspects of the viral life cycle by microscopic techniques. It combines the advantages of genetic labeling suitable for live-cell analyses with the sensitivity and versatility of staining using synthetic fluorescent dyes. The fact that SNAP-tagged molecules can be either stained with various fluorescent dyes or quenched with non-fluorescent substrate allows the application of sequential labeling protocols for pulse-chase approaches [43] which may be applied for the analysis of intracellular Gag trafficking from the site of synthesis. Direct imaging studies of Gag trafficking have so far been hampered by slow maturation kinetics of FPs which impair visualization of newly synthesized Gag molecules. TC-tagged Gag derivatives have been used to circumvent this problem, but studies using this label yielded contradicting results, which might be explained by non-specific intracellular staining with biarsenical dyes [18], [44]. Faster and more specific staining using HIV^SNAP^ could surmount those shortcomings and thereby complement biochemical analyses of Gag distribution. In addition, the possibility to chose between differently colored dyes renders HIV^SNAP^ a versatile tool for multi-color applications, e.g. for investigation of dynamic interactions between HIV Gag and individual host cell factors. Furthermore, the SNAP-tagged viral protein can be labeled with synthetic dyes whose photochemical properties are tailored to the requirements of super-resolution microscopy techniques. In summary, the virus derivative described here opens a variety of possibilities which will allow more detailed investigation of HIV-cell interaction by modern microscopic techniques.

## Materials and Methods

### Plasmids and cell lines

The *snap-tag* coding sequence was amplified by PCR from pSS26m (Covalys) with primers introducing *Cla*I restriction sites at both ends (GGCGCATCGATCCGCCCAGCCCAGGCTTGCC and CAGGGATCGATAGGCGGCATGGACAAAGACTGCGAA, respectively). The resulting fragment was subcloned into pBSMAJ. *Cla*I harbouring a unique *Cla*I restriction site at position 1171 of the NL4-3 sequence [31] resulting in pBSMAJ^SNAP^. A *BssH*II-*Sph*I fragment comprising the modified *MA* sequence was then transferred into the pCHIV and pNLC4-3 [45] context to obtain pCHIV^SNAP^ and pNLC^SNAP^, respectively. For construction of pNLC^iSNAP^, the *snap-tag* coding sequence was amplified by PCR using forward and reverse primers introducing a *Mlu*I and an *Xba*I restriction site, respectively (TGTACAAACGCGTATGGACAAAGACTGC and TTTTGGCTTCTAGAGCCCAGCCCAGGC). The resulting fragment was inserted into pNLC.iGFP, a derivative of pNLC4-3 [45] where an additional HIV protease cleavage site followed by *GFP* had been introduced C-terminally of *MA* by insertion of the *BssH*II-*Sph*I fragment from plasmid HIV Gag-iGFP [33], to obtain pNLC^iSNAP^.

293T [46], HeLa [47], JC53 [48] and TZM-bl [49] cells were grown in Dulbecco's modified Eagle's medium (DMEM GlutaMAX; Invitrogen) supplemented with 100 U/ml penicillin, 100 µg/ml streptomycin and 10% FCS at 37°C, 5% CO_2_. C8166 [50], MT4 [51] and A3.01 [52] cells were kept in RPMI1640 GlutaMAX™ supplemented with 100 U/ml penicillin, 100 µg/ml streptomycin and 10% FCS. Transfections were carried out using polyethyleneimine (PEI) according to standard procedures (293T cells) or FuGeneHD (Roche) according to the manufacturer's conditions. A3.01 T cells were electroporated in a 0.4 cm cuvette (Invitrogen) at voltage 300, capacity of 950 µF in a volume of 500 µl of serum-free medium using the Gene Pulser Xcell System (Bio Rad).

### Particle preparation and immunoblot characterization

293T cells were transfected with the proviral plasmids using PEI. At 44 h post transfection, cells were harvested and lysed in SDS sample buffer. Tissue culture supernatants were precleared by filtration through a 0.45 µM filter and virions were purified by ultracentrifugation through a 20% (w/w) sucrose cushion. Proteins from cell lysates and pelleted particles were separated by SDS-PAGE (acrylamide∶bisacrylamide 200∶1, 17.5% acrylamide) and transferred to a nitrocellulose membrane by semi-dry blotting. Membranes were probed with the indicated primary antisera and bound antibodies were detected by quantitative immunoblot using a LiCor Odyssey system, as well as reagents, protocols and software provided by the manufacturer.

### Analysis of virion infectivity

TZM-bl indicator cells seeded in 96-well plates at 8×10^3^ cells/well were infected one day after seeding using serial dilutions of filtered tissue culture supernatant form 293T cells transfected with pNLC-4.3, pNLC^SNAP^ or pNLC^iSNAP^, respectively. At 48 h post infection, luciferase activity as a measure for infected cells was determined using the SteadyGlo Assay System (Promega) according to the manufacturer's instructions.

### β-Lactamase Virion Fusion Assay

Entry efficiency was determined by a standard HIV fusion assay [34]. Briefly, viral particles were prepared from 293T cells co-transfected with the indicated HIV proviral derivative and pMM310 [53] (plasmid ratio 15∶1 µg). Particle concentration was determined by quantitative immunoblot using antiserum raised against HIV-1 CA. Serial dilutions of virus were used to infect JC53 cells seeded in 96-wells one day prior to infection. Following incubation at 37°C for 6 h, cells were washed with CO_2_-independent DMEM (Invitrogen) and incubated for 12 h at room temperature using CCF2-AM (GeneBLAZER, Invitrogen Ltd., Paisley, UK) according to the manufacturer's protocol. Cells were fixed with 3% PFA/PBS and fluorescence intensities at Em 447 nm and 520 nm (Ex: 409 nm) were determined in a multi-well fluorimeter (Tecan SAFIRE). The ratio of fluorescence emission at 520 nm over emission at 447 nm was calculated and normalized to the corresponding ratio obtained for uninfected cells to yield relative entry efficiencies.

### Replication kinetics of HIV-1 derivatives

HIV, HIV^SNAP^ and HIV^iSNAP^ were produced by transfection of 293T cells with the plasmids pNLC4-3, pNLC^SNAP^ and pNLC^iSNAP^, respectively. The amount of p24 CA in the supernatant was determined by quantitative immunoblot and supernatant corresponding to 0.5 ng of p24 CA was used to infect 1×10^5^ MT-4 cells. Samples of tissue culture supernatant were collected every 2 days the amount of p24 in the supernatant was determined by a quantitative Dot Blot procedure. Briefly, 80 µl of supernatant were spotted onto a nitrocellulose membrane using a Minifold® I device (Whatman). A serial dilution of purified, recombinant HIV-1 CA [54] was used as a standard. The membrane was dried, blocked with 5% skimmed milk powder in TBS/0,5% Tween 20 and probed with rabbit polyclonal antiserum raised against HIV-1 CA. Signals were detected by quantitative immunoblot using a LiCor Odyssey system as well as reagents and protocols provided by the manufacturer and quantitated using Odyssey 2.1 software.

### Intracellular staining of Gag.SNAP

Hela cells seeded on coverslips 24 h prior to the experiment were transfected with FuGene6 (Roche) and incubated at 37°C for 24 h. For SNAP-labelling cells were incubated with 1 µM SNAP-cell TMR-*Star* (New England Biolabs) in DMEM for 15 min at 37°C, washed and incubated in fresh DMEM for 45 min at 37°C to allow diffusion of excess substrate. Cells were then fixed with 3% paraformaldehyde (PFA), counterstained with Hoechst 33258 and embedded in Mowiol. C8166 cells infected with HIV^SNAP^ or HIV were subjected to a modified protocol in solution using the same conditions as above. Between the different steps, cells were pelleted at 4'000 rpm for 4 min. Following fixation with 3% PFA cells were adhered to fibronectin coated cover slips and permeabilized by incubation with 0.1% TritonX-100/PBS and subjected to intracellular staining using rabbit polyclonal serum raised against HIV-1 CA and Alexa488 goat anti rabbit antibody (Invitrogen). Counterstaining of nuclei was performed using Hoechst 33258 in the secondary antibody solution.

### Electron microscopic analysis of cell samples

293T cells were transfected with pNLC4-3 or pNLC^SNAP^, respectively, using FuGene HD (Roche Diagnostics GmbH, Mannheim, Germany). At 24 h post transfection the cells were fixed and processed for resin-embedding as described previously [55]. Sections were examined with a Zeiss EM10 TEM and images taken using a Gatan MultiScan™ camera and Digital Micrograph™ software.

### dSTORM analysis of virions and virus producing cells

Particles were purified from the supernatant of 293T cells transfected with an equimolar mixture of pCHIV and pCHIV^SNAP^ by ultracentrifugation through a 20% sucrose cushion. Particles were adhered to fibronectin coated glass coverslips and fixed with 3% paraformaldehyde in PBS.Viruses were permeabilized with 0.01% Triton X-100 for 10 seconds, blocked with 2% bovine serum albumin (BSA) in PBS for 30 min and stained with 5 µM SNAP-surface 647 (NEB) according to the manufacturer's instructions. For the analysis of HIVexpressing cells, 5×10^5^ A3.01 cells were electroporated with 10 µg of pCHIV^SNAP^. At 24 h post-transfection, cells were transferred to LabTek 8-well chambered coverglasses and fixed with 3% paraformaldehyde in PBS for 15 min. The fixed cells were washed and permeabilized using 0.1% Triton X-100 for 10 min. After blocking with 2% BSA solution for 30 min, cells were stained with 5 µM SNAP-surface 647 according to manufacturer's instructions. dSTORM images were recorded on a custom-built microscope using experimental protocols that were described earlier [41]. Briefly, a multi-line laser (Innova 70C, Coherent, USA) was coupled into an inverted microscope (IX71, Olympus, Japan), and the fluorescence signal was detected using an electron-multiplying charge-coupled device (EMCCD) (Ixon, Andor, Ireland) and appropriate filters and dichroics (AHF, Germany). Photoswitching of Alexa Fluor 647 was realized in oxygen-free aqueous buffer with 100 mM mercaptoethylamine added and using two irradiation wavelengths, 514 nm (0.1–1 kW/cm^2^) for activation and 647 nm (1–5 kW/cm^2^) for read-out. Image reconstruction was performed using the rapidSTORM software package [56]. dSTORM images shown consist of 8000 individual superimposed frames. Summed-molecule TIRF images were reconstructed by summing up single-molecule fluorescence intensities from the complete data set.

## Supporting Information

Figure S1 **Infectivity of HIV^SNAP^ virions after TMR-** ***Star*** **staining.** HIV and HIV^SNAP^ particles were purified by ultracentrifugation from the supernatant of 293T cells transfected with pNLC4-3 or pNLC^SNAP^, respectively, and incubated with 1.2 µM TMR-*Star* or a solvent control (DMSO) for 1 h at room temperature. Unbound dye was removed by gel filtration. (**A**) Western-blot analysis of HIV and HIV^SNAP^ particles stained with TMR-*Star* or DMSO using the indicated antisera. The position of molecular mass standards is indicated at the left (in kDa). (**B**) Staining specificity and efficiency was determined by confocal microscopy of particles adhered to fibronectin coated glass coverslips. TMR-*Star* stained particles were fixed with 3% PFA, permeabilized with 0.01% Triton X-100 and blocked with 2% bovine serum albumin in PBS. HIV-1 CA was detected by immunostaining using a polyclonal sheep anti CA serum and Alexa488 secondary antibody. Representative confocal microscopy images and percentage of signals in the indicated channels are depicted. (**C**) Infectivity of particles as determined by titration of stained (black bars) or solvent treated (white bars) particles on TZM-bl indicator cells as described in Methods. Values were normalized for the amount of input virus as determined by quantitative immunoblot analysis compared to a purified CA protein standard. Mean values and standard deviation of triplicate titrations are shown. RLU, relative light units.
(TIF)

Methods S1 **Infectivity of HIV^SNAP^ particles after staining.**
(DOC)
