# Supplementary figures and images for: A SNAP-Tagged Derivative of HIV-1—A Versatile Tool to Study Virus-Cell Interactions
Source: PLoS One. 2011 Jul 22;6(7):e22007. doi: 10.1371/journal.pone.0022007 (PMC3142126; doi:10.1371/journal.pone.0022007)

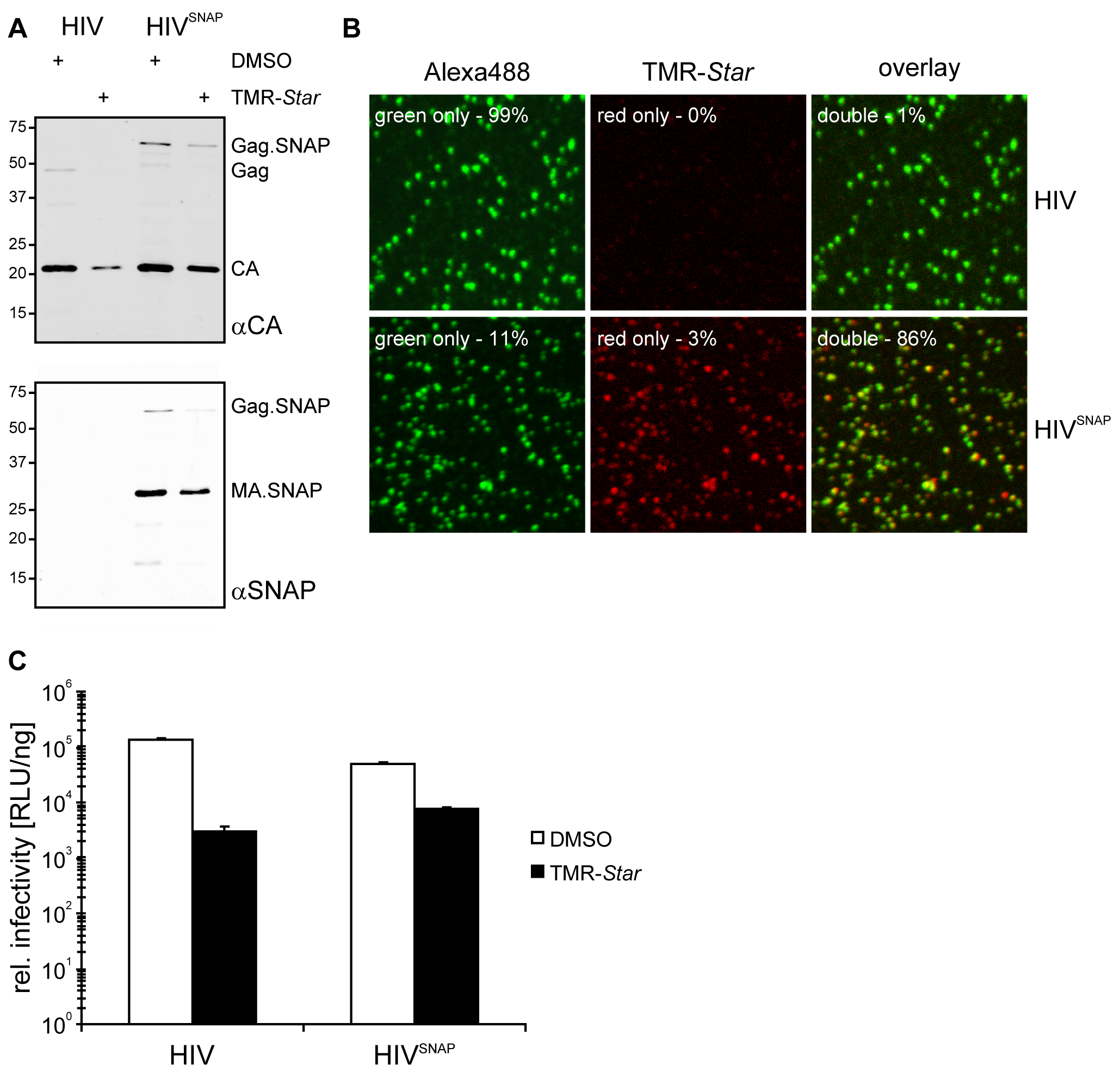

Supplement: Figure S1 — Infectivity of HIVSNAP virions after TMR- Star staining. HIV and HIVSNAP particles were purified by ultracentrifugation from the supernatant of 293T cells transfected with pNLC4-3 or pNLCSNAP, respectively, and incubated with 1.2 µM TMR-Star or a solvent control (DMSO) for 1 h at room temperature. Unbound dye was removed by gel filtration. (A) Western-blot analysis of HIV and HIVSNAP particles stained with TMR-Star or DMSO using the indicated antisera. The position of molecular mass standards is indicated at the left (in kDa). (B) Staining specificity and efficiency was determined by confocal microscopy of particles adhered to fibronectin coated glass coverslips. TMR-Star stained particles were fixed with 3% PFA, permeabilized with 0.01% Triton X-100 and blocked with 2% bovine serum albumin in PBS. HIV-1 CA was detected by immunostaining using a polyclonal sheep anti CA serum and Alexa488 secondary antibody. Representative confocal microscopy images and percentage of signals in the indicated channels are depicted. (C) Infectivity of particles as determined by titration of stained (black bars) or solvent treated (white bars) particles on TZM-bl indicator cells as described in Methods. Values were normalized for the amount of input virus as determined by quantitative immunoblot analysis compared to a purified CA protein standard. Mean values and standard deviation of triplicate titrations are shown. RLU, relative light units. (TIF) [file pone.0022007.s001.tif]
