# Supplementary material for: A SNAP-Tagged Derivative of HIV-1—A Versatile Tool to Study Virus-Cell Interactions
Source: PLoS One. 2011 Jul 22;6(7):e22007. doi: 10.1371/journal.pone.0022007 (PMC3142126; doi:10.1371/journal.pone.0022007)
Supplement: Methods S1 — Infectivity of HIVSNAP particles after staining. (DOC) [file pone.0022007.s002.doc]

**Methods S1**

**Infectivity of HIV^SNAP^ particles after staining**

Particles purified from the supernatant of 293T cells transfected with pNLC4-3 or pNLC^SNAP^, respectively, by ultracentrifugation through a 20% sucrose cushion were resuspended in PBS and incubated with 1.2 µM TMR-*Star* or DMSO, respectively, for 1 h at room temperature. Unbound dye was removed by gel filtration through PD SpinTrap G-25 spin columns (GE Healthcare). Particles were analyzed for the presence of HIV encoded proteins by quantitative immunoblot and for infectivity by titration on TZM-bl indicator cells as described above. To determine the staining efficiency, a sample was adhered to fibronectin coated glass coverslips and fixed with 3% paraformaldehyde. Viruses were permeabilized with 0.01% Triton X-100 for 10 seconds and blocked with 2% bovine serum albumin in PBS for 30 min. HIV-1 CA was detected by immunostaining using a polyclonal sheep anti CA serum and Alexa 488 donkey anti sheep antibody. Image acquisition was performed on a Leica TCS SP2 IRBM confocal laser scanning microscope (Bensheim, Germany) with a HCX PL APO 63x NA 1.4 objective by sequential scan mode.
